# Emphasis on the clinical relationship between alpha-fetoprotein and hepatoid adenocarcinoma of the stomach: a retrospective study

**DOI:** 10.1186/s12876-023-02773-9

**Published:** 2023-05-09

**Authors:** Lamei Li, Xinle Yang, Wei Ji, Qi Zhu, Xin Yang, Junqi Niu, Wanyu Li

**Affiliations:** 1 Department of Infectious Diseases, The Second Affiliated Hospital of Xi’an Jiaotong University, N0.157 Xiwu Road,New Urban District, Xi’an, Shanxi Province, 710004 China; 2 Department of Hepatology, First Hospital, Jilin University, N0.71 Xinmin Street,Chaoyang District, Changchun, 130021 Jilin Provinice China; 3 Department of Oncology, First Hospital, Jilin University, N0.71 Xinmin Street,Chaoyang District, Changchun, 130021 Jilin Provinice China

**Keywords:** Hepatoid adenocarcinoma of  stomach, Clinicopathological characteristics, Prognosis, AFP level

## Abstract

**Background:**

Hepatoid adenocarcinoma of the stomach (HAS) is a highly malignant and rare extrahepatic tumor. The prognosis is controversial because of its rarity and the lack of multi-center cohort studies, especially on the influence of serum Alpha-fetoprotein (AFP) level on prognosis. We aimed to analyze the clinicopathological characteristics and prognosis of HAS, particularly the effect of serum AFP on the prognosis of HAS.

**Methods:**

We retrospectively reviewed clinical data of one HAS patient treated at our institution in 2019 and of 252 patients reported between 1984 and 2020 in research databases.

**Results:**

Among these patients, 60.1% were > 60 years, 51% had lesions in the gastric antrum, and 51.0% (73/143) had the ulcerative lesion type. The preoperative elevated levels of serum alpha-fetoprotein (AFP) were detected in most patients (76.7%). Lymph-node (84.6%) and preoperative liver metastasis (39.1%) were often found. The high-AFP group was characterized by a higher rate of stage IV (*P* = 0.000682) and liver metastasis (*P* = 0.000068). The 1-, 3-and 5-year progression-free survival(PFS) rates were 41%, 18%, and 0%, and the 1-, 3-, and 5-year overall survival (OS) rates were 64%, 26%, and 21%, respectively. The survival analysis showed that OS was significantly shorter for HAS with high-AFP (> 300 ng/ml) than with low-AFP (≤ 300 ng/ml) (*P* = 0.023). The univariate analysis indicated that the OS of HAS was associated with tumor location, pTNM stage, lymph-node metastasis, surgical resection, and serum AFP > 300 ng/ml. However,the prognostic factors for PFS was only pTNM stage and surgical resection. The multivariate analysis confirmed that the independent prognostic factor affecting OS of HAS included pTNM stage and surgical resection.

**Conclusions:**

Liver metastasis was increasingly more likely with increasingly higher serum AFP, but the prognosis of HAS is not necessarily poor. Serum AFP level is an important prognostic indicator in HAS and should be monitored.

## Background

Hepatoid adenocarcinoma (HAC) is a highly malignant and rare extrahepatic tumor histologically similar to hepatocellular carcinoma (HCC) and characterized by high serum α-fetoprotein (AFP). Additionally, HAC has been reported in various organs, such as the esophagus, stomach, colon, lung, pancreas, peritoneal, and ovaries, but gastric tissue is the most common [1-6]. In 1970, the first case of AFP-producing gastric cancer with liver metastasis was reported by Bourreille J et al. [7]. In 1985,Ishikura et al. [8] formally proposed the term “hepatoid adenocarcinoma of the stomach (HAS)”,which is the primary gastric cancer characterized by liver-like differentiation and very high production of massive α-fetoprotein (AFP) that is detected in the serum.

HAS is a unique and rare subtype of gastric adenocarcinoma, accounting for 0.38%–1% of all gastric cancers [9]. Many reports of HAS about clinical manifestations and pathological features as well as some studies about prognosis have been published since the research of Ishikura et al., but most studies were limited to single-case reports or case series. Because of the rarity of HAS and lack of multi-center cohort studies, there are many problems with prognosis, in particular, the effect of serum AFP levels on prognosis is highly controversial.Inoue et al. [10] found that serum AFP levels were not associated with the prognosis of HAS, but H. J. Lin [11] reported poorer prognosis in HAS patients with elevated serum AFP than with normal serum AFP, and AFP > 300 ng/ml was associated with poor prognosis.Since several published articles on prognosis used only univariable factors for analysis,few articles have included serum AFP > 300 ng/ml in multivariable analysis.

The study aim was to analyze the clinicopathological characteristics and prognosis of HAS, particularly the effects of serum AFP level on prognosis.

## Methods

### Patient population

**Fig. 1:**
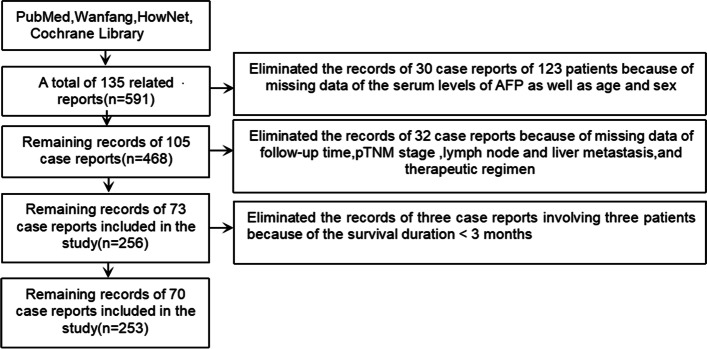
Flow chart showing the study data-collection process

In this study,we used the keywords “hepatoid adenocarcinoma,” “stomach,” and “AFP” to retrieve single cases or case series from Same-Center published from 1984–2020 in the four major databases (PubMed, HowNet, Wanfang, Cochrane Library).Patients clearly diagnosed as HAS by surgery or gastroscopy biopsy with a tumor located in the stomach were included in this study, and patients with cirrhosis or HCC were excluded. Clinical data and follow-up times were extracted from published case reports. We have reclassified the pathology-Tumor-Node-Metastasis (pTNM) stage of all tumors according to the 7^th^ edition of the American Joint Committee on Cancer. Finally, we identified 135 reports (including 591 patients and one patient from our hospital diagnosed as HAS in 2019). Thirty reports (including 123 patients) were excluded because of the lack of information about age, sex, or serum AFP levels. Since our study mainly discussed the prognosis of HAS patients, 32 of the remaining 105 reports were eliminated because they provided no information on follow-up time,survival status,pTNM staging,lymph-node metastasis,preoperative liver metastasis,and treatment protocol.The follow-up period ranged from 3 to 131.8 months and three patients were removed for < 3 months (one died 2 weeks after surgery,one died 3 weeks after surgery, and the other died 50 days after surgery). Ultimately,253 patients in the 70 reports were included in the study (Fig. 1).

### Pathological diagnosis

Pathology is the “gold standard” for diagnosing the HAS,morphology is the main basis for pathological diagnosis.Gastric cancer with hepatoid differentiation area in pathomorphology can be diagnosed as HAS regardless of the number of histological hepatoid differentiation [12]. The tumor cells generally form two areas: adenocarcinoma and hepatocellular cancer,the migration among the two areas.In the hepatocellular cancer area,the cancer cells are large, irregular in shape with abundant cytoplasm, the cytoplasm exhibits pale eosin staining,and form the medullary or cable structure,which are divided into by a few fibrous tissues and a rich blood supply [13, 14]. Immunohistochemical(IHC) staining is helpful for the diagnosis of HAS,More than 90% of HAS express AFP, which is often positive or strongly positive in the hepatoid differentiation area, and generally negative in the common adenocarcinoma area. However, positive IHC AFP staining is not necessary for the diagnosis of HAS.

### Statistical analysis

The assessment of clinicopathological data was performed by using IBM SPSS Statistics for Windows version 26.0 software (IBM Corp. Armonk, NY, USA). Comparisons of the categorical variables were analyzed by using either the Chi-square test or Fisher’s exact test, as appropriate. Numerical variables were expressed as the mean ± standard deviation. The Kaplan–Meier method was used to evaluate both the overall survival (OS) and progression-free-survival (PFS), with the log-rank test being used to examine any differences between survival curves. The Cox regression models were used to determine prognostic factors associated to HAS,variables with *P* value less than 0.1 in the univariate model were included for further identify significant independent predictors of survival of HAS in the multivariable model.OS was calculated from the date of pathological diagnosis to the date of death or end date of follow-up, and PFS was determined from the date of surgery resection or chemotherapy to the date of recurrence or metastasis of the primary tumor. *P* < 0.05 was taken to be indicative of statistical significance for all tests. Kaplan–Meier survival plots were made by using GraphPad Prism 8 (San Diego, CA, USA).

## Results

### Patient characteristics

The available clinicopathological characteristics of the 253 HAS patients are summarized in Table 1. The patients ranged in age from 35 to 83 years (median, 63 years, mean, 61.98 years), 60.1% were > 60 years old, 75.1% were male, and the male:female ratio was 3:1. The tumors were located in the gastric antrum in 51.4% of the patients, 134 (134/167, 80.2%) were poorly differentiated, and the most common lesion was the ulcerative type (73/143, 51.0%). Most patients had reached the advanced stage when diagnosed, and pTNM staging ranged from I–IV in 3.2%, 13.4%, 29.2%, and 54.2%, respectively. Of the 60 patients with information on the depth of infiltration of the gastric wall, cancer cells penetrated serosa layer and beyond in 75% (45/60) patients.The majority of patients (76.7%) had elevated serum AFP levels, and 53% had serum AFP ≥ 300. The value of AFP decreased in 81.4% (92/113) of patients after surgery, but AFP increased again with tumor recurrence or metastasis. AFP increased after surgery in 18.6% of the patients, lymph-node metastasis occurred in 84.6%, and liver metastases were present in 39.1% before surgery. A total of 226 patients underwent surgical resection, and 27 patients did not undergo surgery.

**Table 1:** Baseline characteristics of 253 patients with HAS

Characteristics	*n* (%)
Age (year)*,* median = 63 (35–83)	
≤ 60	101 (39*.*9)
> 60	152 (60.1)
Sex	
Male	190 (75*.*1)
Female	63 (24*.*9)
Location	
Antrum	130 (51*.*4)
Nonantrum	123 (48*.*6)
Differentiation (167)	
Poorly differentiated	134 (80.2)
Moderately and well differentiated	33 (19*.*8)
Lesion type (143)	
Ulcerative/upheaval-serrated type	73 (51*.*0) / 3(2*.*1)
Upheaval type	20 (14*.*0)
Infiltrating and upheaval–infiltrating type	47 (32*.*9)
pTNM stage (253)	
I	8 (3*.*2)
II	34 (13*.*4)
III	74 (29*.*2)
IV	137 (54*.*2)
Depth (60)	
T1–T2	15 (25*.*0)
T3–T4	45 (75*.*0)
AFP of serum (ng/ml)	
≤ 300 (ng/ml)	134 (53 0)
> 300 (ng/ml)	119 (47 0)
Metastasis	
Lymph-node metastasis	214 (84.6)
Preoperative liver metastasis	99 (39*.*1)
Surgical resection	226 (89*.*3)

Dates were displayed in counts(%)

*pTNM* Pathology-Tumor-Node-Metastasis, *AFP* Alpha-Fetoprotein

### Significance of AFP level in HAS

To further study the clinical significance of AFP levels in HAS, according to serum AFP with a cutoff value of 300 ng/ml, we divided HAS into the low-AFP group (serum AFP ≤ 300 ng/ml) and high-AFP group (serum AFP > 300 ng/ml) to further compare the clinicopathological features of the two groups (Table 2). The results showed that there were no significant differences in sex, age, tumor differentiation degree, and lymph-node metastasis. When compared with the low-AFP group, the tumors in the high-AFP group were more likely to occur in the gastric antrum, and the most common lesions were the ulcerative type. The most common stage at the initial diagnosis was pTNM IV (*P* < 0.001). Additionally, the incidence of liver metastasis was higher (37.4% vs. 62.6%) and tumor size was smaller in the high-AFP group. We observed that high-AFP tumors were often limited to the mucosa, submucosa, or muscle layer, whereas low-AFP tumors often invaded the serosa layer and beyond.

**Table 2:** Comparison of clinicopathological characteristics between patients with HAS with low and high AFP

Parameters	AFP concentration		*X*^*2*^	*P*^*a*^
	** ≤300 (ng/ml);** ***n *** **(%)**	**≤300 (ng/ml);** ***n *** **(%)**		
Sex			2.692	0.101
Male	95 (50.0)	95 (50.0)		
Female	39 (61*.*9)	24 (38*.*1)		
Age (years)			0.016	0.899
≤ 60	53 (52*.*5)	48 (47*.*5)		
> 60	81 (53*.*3)	71 (46*.*7)		
Differentiation (167)			0.788	0.375
Poorly differentiated	78 (58*.*2)	56 (41*.*8)		
Moderately/well differentiated	22 (66*.*7)	11 (33*.*3)		
Lymph-node metastasis			2*.*296	0.13
No	25 (64*.*1)	14 (35*.*9)		
Yes	109 (50.9)	105 (49*.*1)		
Preoperative liver metastasis			15*.*869	** < 0.001**
No	97 (63*.*0)	57 (37*.*0)		
Yes	37 (37*.*4)	62 (62*.*6)		
Location			3*.*918	**0.048**
Antrum	61 (46*.*9)	69 (53*.*1)		
Notantrum	73 (59*.*3)	50 (40.7)		
Lesion type (143)			16*.*371	** < 0.001**
Ulcerative type	30 (41*.*1)	43 (58*.*9)		
Upheaval type	12 (60.0)	8 (40.0)		
Infiltrating/upheaval-infiltrating type	35 (74*.*5)	12 (25*.*5)		
Upheaval-serrated type	0 (0.0)	3 (100.0)		
pTNM stage			17*.*247	** < 0.001**
I	4 (50.0)	4 (50.0)		
II	21 (61*.*8)	13 (38*.*2)		
III	52 (70.3)	22 (29*.*7)		
IV	57 (41*.*6)	80 (58*.*4)		
Tumor size (90)			4*.*275	**0.039**
≤ 5 cm	21 (38*.*9)	33 (61*.*1)		
> 5 cm	22 (61*.*1)	14 (38*.*9)		
Depth (60)			5*.*714	**0.017**
T1–T2	3 (20.0)	12 (80.0)		
T3–T4	25 (55*.*6)	20 (44*.*4)		

Statistically significant values are shown in bold

X^2^ Chi-square value

Dates were displayed in counts(%);

*AFP* Alpha-Fetoprotein, *pTNM* Pathology-Tumor-Node-Metastasis

^a^Chi-square test

### Survival and prognosis

**Fig. 2:**
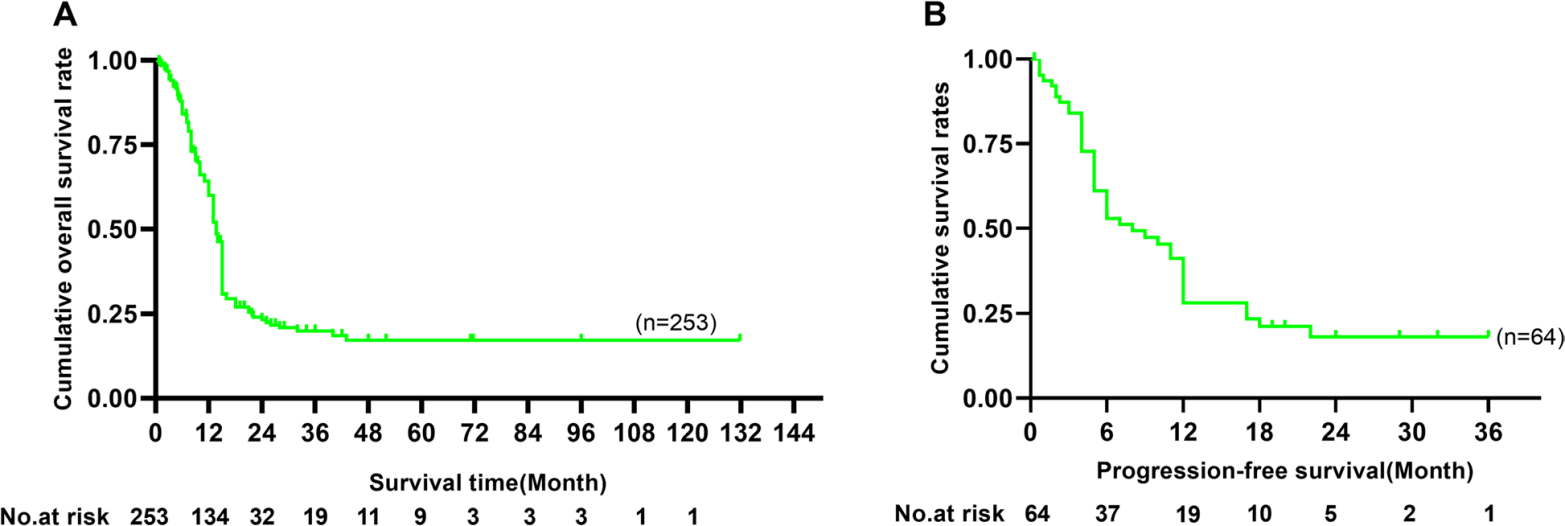
The cumulative overall survival rates of 253 patients and cumulative recurrence rates of 64 patients with HAS*.* **A** the OS rates were 64% at 1 year, 26% at 3 years, and 21% at 5 years; **B** the PFS rates were 41% at 1 year, 18% at 3 years, and 0% at 5 years

**Fig. 3:**
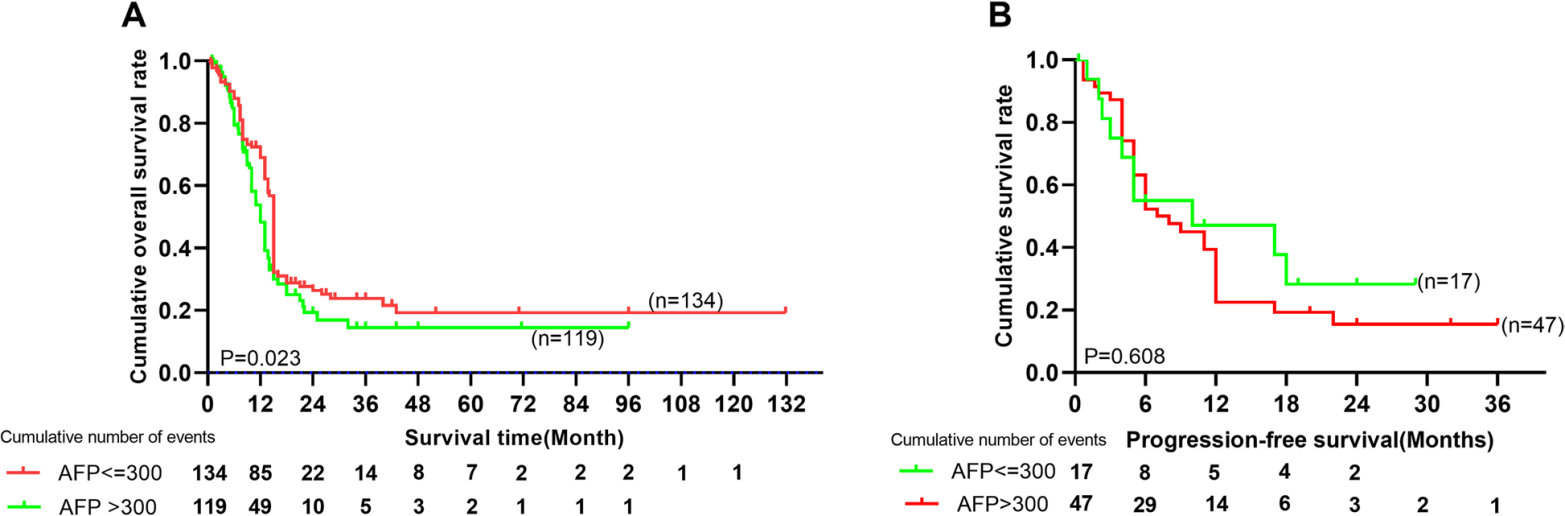
Comparison of the OS rates of 253 patients and progression-free survival rates of 64 patients between the patients with AFP ≤ 300 ng/ml and those with AFP > 300 ng/ml in HAS*.* OS was poorer for patients with HAS in the high-AFP group than those in the low-AFP group (*P* = 0.023), but the difference was not statistically significant regarding PFS (*P* = 0.608). The 1-, 3-, and 5-year OS rates were 72% vs. 56%, 30% vs. 22%, and 25% vs. 16% in the low-AFP vs. high-AFP groups

Survival data of 253 patients were finally included in the study for analysis OS. The data of only 64 patients were used to analyze PFS, because 189 patients lacked information about the first recurrence or metastasis after surgery or chemotherapy. The follow-up time ranged from 3.0–131.8 months, the median survival time for OS was 12 months (range: 0.7–131.8 months), and the median PFS time was 6 months (range: 0.3–36 months). The 1-, 3-, and 5-year PFS rates were 41%, 18%, and 0%, respectively, and the 1-, 3-, and 5-year OS rates were 64%, 26%, and 21%, respectively (Fig. 2). The survival analysis showed that OS was significantly poorer for HAS patients in the high-AFP group than in the low-AFP group (*P* = 0.023), but the difference was not statistically significant in terms of PFS in these two groups. The 1-, 3-, and 5-year OS rates were 72% vs. 56%, 30% vs. 22%, and 25% vs. 16% (*P* = 0.023) in the low-AFP and high-AFP groups (Fig. 3). A total of 45 (70.3%) patients experienced recurrence or metastasis, and 161 (63.6%) patients died.

### Risk factors for prognosis

Univariate analysis results indicated that tumor location, pTNM stage, lymph-node metastasis, surgical resection, and serum AFP > 300 ng/ml were associated with the OS of HAS(*P* < 0.05).However,the prognostic risk factors for PFS only included pTNM stage and surgical resection. The multivariable analysis confirmed that pTNM staging [hazard ratio (HR) = 1.406, 95% confidence interval (CI): 1.085–1.822, *P* = 0.010] and surgical resection (HR = 0.437, 95% CI: 0.256–0.746, *P* = 0.002) were independent prognostic factors affecting OS of HAS.However, surgical resection (HR = 0.267), 95% CI: 0.098–0.729, *P* = 0.010) was only independent prognostic factor for PFS because of the limited number of patients (Tables 3 and 4).

**Table 3:** Univariate and multivariate analysis of OS in 253 patients with HAS

Parameters	Univariate			Multivariate		
	**HR**	**95% CI**	***P***	**HR**	**95%CI**	***P***
Sex	1.080	0.762*–*1.531	0.664			
Age	0.847	0.619*–*1*.* 159	0.299			
Location	1.488	1.086–2.039	**0.013**	1.394	1.003–1.937	0.048
pTNM stage	1*.* 489	1*.* 219*–*1*.* 818	** < 0.001**	1*.* 406	1*.* 085*–*1*.* 822	**0.010**
Liver metastasis (vs. no)	1*.* 358	0.984*–*1*.* 876	0.063	0.790	0.528*–*1*.* 182	0.251
Lymph*-*node metastasis (vs. not)	1*.* 870	1*.* 150*–*3*.* 043	**0.012**	1*.* 474	0.892*–*2*.* 436	0.130
Surgery (vs. not)	0.375	0.230–0.612	** < 0.001**	0.437	0.256–0.746	**0.002**
AFP (≤ 300 ng/ml/ ≥ 300 ng/ml)	1*.* 435	1*.* 050*–*1*.* 960	**0.023**	1*.* 230	0.887*–*1*.*706	0.215

Statistically significant values are shown in bold

*pTNM* Pathology-Tumor-Node-Metastasis, *AFP* Alpha-Fetoprotein, *HR* Hazard Ratio, *95%CI* 95% confidence interval

**Table 4:** Univariate and multivariate analysis of DFS in 64 patients with HAS

parameters	Univariate			Multivariate		
	**HR**	**95% CI**	***P***	**HR**	**95%CI**	***P***
Sex	0.974	0.492–1.929	0.941			
Age	0.695	0.382–1.265	0.234			
Location	0.978	0.540–1.771	0.941			
pTNM stage	1.578	1.035–2.407	**0.034**	1.433	0.912–2.253	0.119
Liver metastasis(vs. not)	1.317	0.684–2.539	0.410			
Lymph node metastasis(vs. not)	2.455	0.874–6.899	0.088	2.255	0.795–6.402	0.127
Surgery(vs. not)	0.208	0.078–0.557	**0.002**	0.267	0.098–0.729	**0.010**
AFP (≤ 300 ng/ml/ ≥ 300 ng/ml)	1.192	0.587–2.421	0.628			

Statistically significant values are shown in bold

*pTNM* Pathology-Tumor-Node-Metastasis, *AFP* Alpha-Fetoprotein, *HR* Hazard Ratio, *95%CI* 95% confidence interval

## Discussion

Although HAS can be characterized by AFP secretion, there has been no obvious evidence of the ability of AFP to predict prognosis, mainly because of limited reports and small samples in previous studies. In our study, HAS was more likely to affect individuals in their 60 s, showed high male predominance, was most commonly located in the gastric antrum, and was most commonly the ulcerative lesion type, findings that were consistent with those of previous studies [15, 16]. Similar to the clinical symptoms of ordinary gastric cancer, those of HAS are atypical, the onset is insidious, most tumors have reached the advanced pTNM stage when diagnosed, and the highly aggressive nature is associated with poor prognosis. HAS is often accompanied by an increase in serum AFP, possibly because the stomach and liver originated from the endodermal primitive anterior intestine during embryonic development, as previously reported [17]. In this study, 76.7% of patients had an elevated AFP. The serum AFP level generally decreased after surgery in the majority of patients, but it increased again after recurrence or metastasis. Serum AFP level can be used to monitor tumor recurrence or metastasis. According to previous reports, the incidence of liver metastasis of HAS was 38.1–41.9% [12, 18], which is consistent with the incidence found in our study.

Considering that AFP level plays an important role in the diagnosis and treatment of HAS, we performed a subgroup analysis of serum AFP with a cutoff value of 300 ng/ml. Consistent with previous reports, the high-AFP group was characterized by a higher rate of stage IV and liver metastasis [11, 19], possibly because AFP has an immunosuppressive function in the human body. In the presence of AFP, cancer cells grow faster and can form cancer bolts by invading the vasculature, resulting in distant metastasis [20]. Surprisingly, we found that the low-AFP group had larger tumors, and tumors often invaded the serosa layer and beyond, which was contrary to previous assumptions. There are possibly three reasons for this difference: (1) AFP-producing gastric cancer has been described in some studies [11]; (2) differences in AFP cutoff values [21]; (3) small sample size. More investigation with a larger sample size will be required to verify these findings and draw accurate conclusions.

According to previous knowledge, the prognosis of HAS was worse than that of common gastric cancer [21]. Our study found that high-AFP was not associated with the PFS of HAS, whereas the high-AFP group had significantly poorer OS, findings that were consistent with the findings of Er-Bao Chen et al. [11, 12, 19, 22]. There is no clear molecular mechanism explanation for the poor prognosis of HAS. Some scholars believe that HAS can produce alpha-1 anti-trypsin (AAT) and/or alpha-1 anti-chymotrypsin (ACT) and AFP. AAT and ACT have immunosuppressive and protease inhibitory properties, which can enhance invasiveness. In addition, AFP has an inhibitory effect on lymphocyte transformation [23, 24].

Because HAS tended to progress to lymph-node metastasis, preoperative distant metastasis, and vascular invasion, it has a poor prognosis. Related literature has reported that the average survival period of HAS was 10–18 months, and we found that the median survival time for OS was 12 months [25-27]. Due to selection bias and recall bias in retrospective studies, the results of multivariate analyses have varied among different reports. Some research has indicated that independent prognostic factors of HAS included pTNM stage, liver metastasis, and surgical resection, among others [18, 28, 29]. A study by H. J. Lin [11] found that high-AFP level was also an independent risk factor of HAS. Our study showed that tumor location, lymph-node metastasis, pTNM stage,and high-AFP level were associated with poor prognosis of HAS, whereas multivariable analysis confirmed that surgical resection could effectively prolong survival and reduce primary tumor recurrence or progression.We speculate that early diagnosis of HAS and surgical resection may be critical to improve prognosis.

Therefore, in clinical practice, HAS should be highly suspected in patients with gastric adenocarcinoma with elevated AFP, and a pathological biopsy was performed as early as possible to obtain a definite diagnosis. Once diagnosed with HAS, resection should be the first choice if conditions permit. We concluded that surgery can improve the survival rate of patients with HAS. However, for those patients who have missed the opportunity for surgery at the time of diagnosis or who relapse after surgery or chemotherapy, relevant research has reported that immunotherapy combined with chemotherapy and (or) targeted therapy might be beneficial in HAS patients [30-33].

Our study had some limitations that should be considered. First, this was a retrospective study of published case reports, and the clinical data are incomplete. Second, the sample for analyzing PFS was not sufficiently large, which may have caused some statistical bias. Therefore, a multi-center cohort study with a larger sample size is needed to verify our findings.

## Conclusions

The majority of patients with HAS have elevated serum AFP, and the higher the serum AFP, the more likely liver metastasis will occur. However, the prognosis of HAS is not necessarily poor. High-AFP (> 300 ng/ml) was significantly associated with poorer OS in HAS, so physicians should monitor serum AFP in clinical practice. Early pTNM staging and surgical resection can improve the prognosis of patients with HAS.

## Data Availability

All data generated or analyzed during this study are available from the first author on reasonable request.

## Abbreviations

AFP: Alpha-Fetoprotein
pTNM: Pathology-Tumor-Node-Metastasis
OS: Overall Survival
PFS: Progression-Free Survival
HR: Hazard Ratio
95%CI: 95% Confidence interval
